# Reply to Lau et al Re: “Antipseudomonal Antibiotics in Diabetic Foot Infections: A Practical Perspective From a Community Hospital”

**DOI:** 10.1093/ofid/ofae259

**Published:** 2024-06-26

**Authors:** Michael P Veve, Nimish Patel

**Affiliations:** Department of Pharmacy, Henry Ford Hospital, Detroit, Michigan, USA; Department of Pharmacy Practice, Eugene Applebaum College of Pharmacy and Health Sciences, Wayne State University, Detroit, Michigan, USA; Division of Clinical Pharmacy, Skaggs School of Pharmacy and Pharmaceutical Sciences, University of California at San Diego, La Jolla, California, USA

To the Editor—Lau and colleagues evaluated diabetic foot infections (DFIs) at a community hospital in Washington State, United States and observed a high use of antipseudomonal antibiotics despite a low number of cultures with *Pseudomonas aeruginosa* growth [1]. These findings add to the existing literature with the inclusion of DFIs with bony involvement. Furthermore, these data are consistent with our prior multicenter work of individuals with nonbony DFIs and other data that highlight the lack of change in overprescribing antipseudomonal therapy in DFI [2–4].

One of the conclusions from Lau and colleagues is that “initiating antipseudomonal therapy based on risk factors alone might still lead to unnecessarily broad antimicrobial coverage” [1]. While we can generally agree with this comment, identification and application of patient-specific risk factors are a foundational antimicrobial stewardship concept. We feel that the same challenges brought up by Lau and colleagues could be extrapolated to most infectious disease states where organism risk factor analyses have been performed. Our prior work identified immunocompromising conditions and outpatient DFI treatment failure within 90 days as having associations with *P aeruginosa* and are representative of patient data from 5 distinct geographic locations in the United States [2]. It is also important to note that risk factors are only one component that goes into the decision to use broad-spectrum antibiotics for individuals presenting with a DFI. Other factors to consider are the quality of diagnostics, source control, local epidemiologic data, and other patient-specific exposures that could lead to a *P aeruginosa* infection. Regardless, the incidence of *P aeruginosa* DFIs is indeed very low. We believe that stewardship efforts are best spent holistically evaluating patients, designing pragmatic interventions, and providing education that promotes optimal DFI therapy. This could include a local risk factor analysis, standardized procedures for obtaining quality cultures/initiating source control, and/or modifying local empiric DFI treatment guidelines to suggest narrow-spectrum antibiotic therapy.

It is interesting to note that in the cohort of patients who were hospitalized for DFI that Lau and colleagues evaluated, only 28% presented with sepsis or systemic symptoms [1]. This is also our anecdotal experience when treating patients and suggests an opportunity to hold empiric antibiotic therapy until a quality skin, tissue, or bone culture is obtained. Similarly, this practice is recommended in patients without signs of systemic infection in patients with native vertebral osteomyelitis [5]. While this approach can prevent unnecessary and potentially harmful therapy, our experience suggests that it can be challenging to implement in hospital settings and for DFIs. Another point to consider is that the methodology used to identify pathogens associated with DFIs has historically been limited to microbiologic culture and susceptibility testing. The total processing time for this approach can be up to 3 days and still yield no pathogen. This highlights the need to investigate emerging and unbiased technologies that could improve pathogen detection, such as metagenomic sequencing.

The pathophysiology of DFIs makes them inherently challenging to manage with antibiotic therapy alone; poor tissue perfusion and blood flow to foot tissue or bone lead to inadequate antibiotic concentration at the target site, with subsequent treatment failure or recurrent infection [3]. Perhaps one of the most valuable DFI interventions that should be considered is early consultation with wound care, surgery, and/or orthopedic specialists in scenarios where bone involvement is present. In our opinion and that of the International Working Group on the Diabetic Foot, performing timely surgical intervention/source control represents the mainstay of DFI management in persons presenting with moderate to severe diabetes-related foot infections, which is a similar conclusion made by Lau and colleagues [1, 6–9]. Additionally, consulting these individuals can result in obtaining a quality surgical culture that is better apt to guide antibiotic treatment [10]. Future research could focus on the impact of these individuals on DFI outcomes as a “DFI care bundle,” including consultation timeliness and intensity of wound care or surgical intervention.

Additionally, future DFI research directions could include prospective evaluation of moderate or severe infections with randomization to empiric broad-spectrum or targeted narrow-spectrum antibiotic therapy based on relevant patient history, key physical findings, and available culture or local antibiotic susceptibility data. These data would be complementary to existing randomized control trial data such as SIDESTEP, which compared ertapenem with piperacillin-tazobactam in moderate to severe DFIs [11].

It is also important to discuss that while *P aeruginosa* is an uncommonly cultured organism in persons with DFIs in the United States, it is a much more frequent pathogen in warm/tropical countries based on data from the Middle East, North Africa, India, Southeast Asia, and other areas [3, 12–15]. Factors that may alter these populations’ risk to infection with this water-borne organism can include sweating feet, often put into water-holding leather sandals; the practice of feet washing multiple times a day among some Muslim populations [16]; and easy access to over-the-counter antibiotics in many of these countries.

Given that DFIs are so prevalent in the United States, we feel that this disease state represents a significant opportunity for antimicrobial stewardship intervention. More data are needed that describe successful strategic interventions in DFIs that result in avoiding unnecessary antipseudomonal therapy. We encourage Lau and colleagues to pursue eventual publication of their recent efforts in promoting narrower-spectrum empiric DFI coverage as a quasi-experiment once their intervention has matured.

## Note

***Potential conflicts of interest*.** All authors: No reported conflicts.
